# Etomoxir: an old dog with new tricks

**DOI:** 10.1016/j.jlr.2024.100604

**Published:** 2024-08-02

**Authors:** Reagan M. McGuffee, Kyle S. McCommis, David A. Ford

**Affiliations:** Edward A. Doisy Department of Biochemistry and Molecular Biology and Center for Cardiovascular Research, Saint Louis University School of Medicine, St. Louis, MO, USA

Mitochondrial β-oxidation of fatty acids is critical for cellular energetics and has been a targeted pathway for many diseases including type-2 diabetes, ischemic heart disease, liver disease, and cancers. Although organs and tissues are metabolically flexible, many preferably depend on mitochondrial fatty acid β-oxidation for their energetic needs. For example, approximately 70% of myocardial ATP is derived from fatty acid β-oxidation. Fatty acid β-oxidation requires initial extra-mitochondrial fatty acyl CoA production followed by its conversion to fatty acylcarnitine mediated by carnitine palmitoyl transferase 1 (CPT1) to enter the mitochondria. CPT1 is the rate-limiting step for mitochondrial β-oxidation and is inhibited endogenously by malonyl CoA, the product of the rate-limiting enzymatic step of fatty acid synthesis, acetyl CoA carboxylase. There are three isozymes of CPT1 (1). CPT1A is predominantly found in liver. CPT1B is predominant in muscle, including the heart. CPT1C is abundant in the brain. CPT1C has reduced catalytic activity in comparison to CPT1A and CPT1B although it may have important nutrient-sensing roles (2). Interestingly, CPT1B is 30- to 100-fold more sensitive to malonyl CoA than CPT1A (3). Due to the key role of CPT1 in mitochondrial fatty acid β-oxidation, pharmacological inhibitors of CPT1 have been used as tools by lipid researchers investigating the role of β-oxidation in disease. For example, limiting β-oxidation by CPT1 inhibition has been shown to shift metabolism from fatty acid-dependence to glucose-dependence following myocardial ischemia, type-2 diabetes, and various cell cancers (4).

There are two commonly used types of CPT1 inhibitors including glycidic acid analogs like etomoxir (ETO) and tetradecyl glycidic acid and the aminocarnitine derivative, ST1326 (1). ETO is a commonly used inhibitor that does not have isozyme selectivity. ETO underwent phase 2 clinical trials for congestive heart failure, which revealed it caused hepatotoxicity (5). ETO inhibition of CPT1 is dependent on its conversion to ETO-CoA (6). ETO-CoA contains the oxirane ring of ETO, which opens and covalently modifies CPT1 (6). The IC_50_ for ETO varies from tissue to tissue and species to species. For example, there is 100-fold increase in CPT1 sensitivity to ETO comparing human versus rat hepatocytes (7). The disparate levels of ETO needed to inhibit CPT1 are complicated by off-target effects that have been observed at higher concentrations (8). For example, above 5 μM, ETO causes severe oxidative stress in T-cells (9). Collectively, these findings highlight that off target effects and the mechanisms responsible for off-target effects need to be considered with the use of ETO.

In this issue of JLR, Richard Gross’s group sheds new light on mechanisms responsible for ETO inhibition of CPT1 as well as mechanisms leading to off-target effects. Using mass spectrometry to assess acylcarnitine production by CPT1 in heart and liver mitochondria, they observed an unexpected monochlorinated, carnitine-containing compound in mitochondria treated with ETO. This compound was carefully characterized to reveal it was the carnitine derivative of ETO (ETO-carnitine) (Fig. 1). They demonstrated the conversion of fatty acids to acylcarnitines by intact murine heart mitochondria was inhibited by ETO with an IC_50_ of 1.4 μM, which also mirrored the concentration-dependent production of ETO-carnitine. ETO-carnitine was subsequently found to retain the oxirane ring, which is essential for the covalent modification of CPT1 by ETO-CoA. Based on the nucleophilic attack of the oxirane ring of ETO-CoA by serine in CPT1 and the outer mitochondrial membrane permeability of ETO-carnitine, they then suggested ETO-carnitine could covalently modify through nucleophilic attack of the active site serine residue of iPLA_2_β and iPLA_2ϒ_, which the Gross group had previously demonstrated reside, in part, in mitochondria. These phospholipases were extremely sensitive to ETO-carnitine with IC_50_ < 2 nM. In contrast, the IC_50_ for cPLA_2_α was greater than 40 nM, though it was not confirmed whether covalent modification and/or a high structural similarity to phosphatidylcholine substrates contributed to the inhibition. Lastly, ETO-carnitine was shown to inhibit fatty acid oxidation in a CPT1-independent manner by measuring the inhibition of mitochondrial respiration with palmitoylcarnitine as substrate, which does not require CPT1 (Fig. 1). Whether this inhibition of respiration is related to iPLA_2_ inhibition by ETO-carnitine remains unknown.

**Fig. 1:**
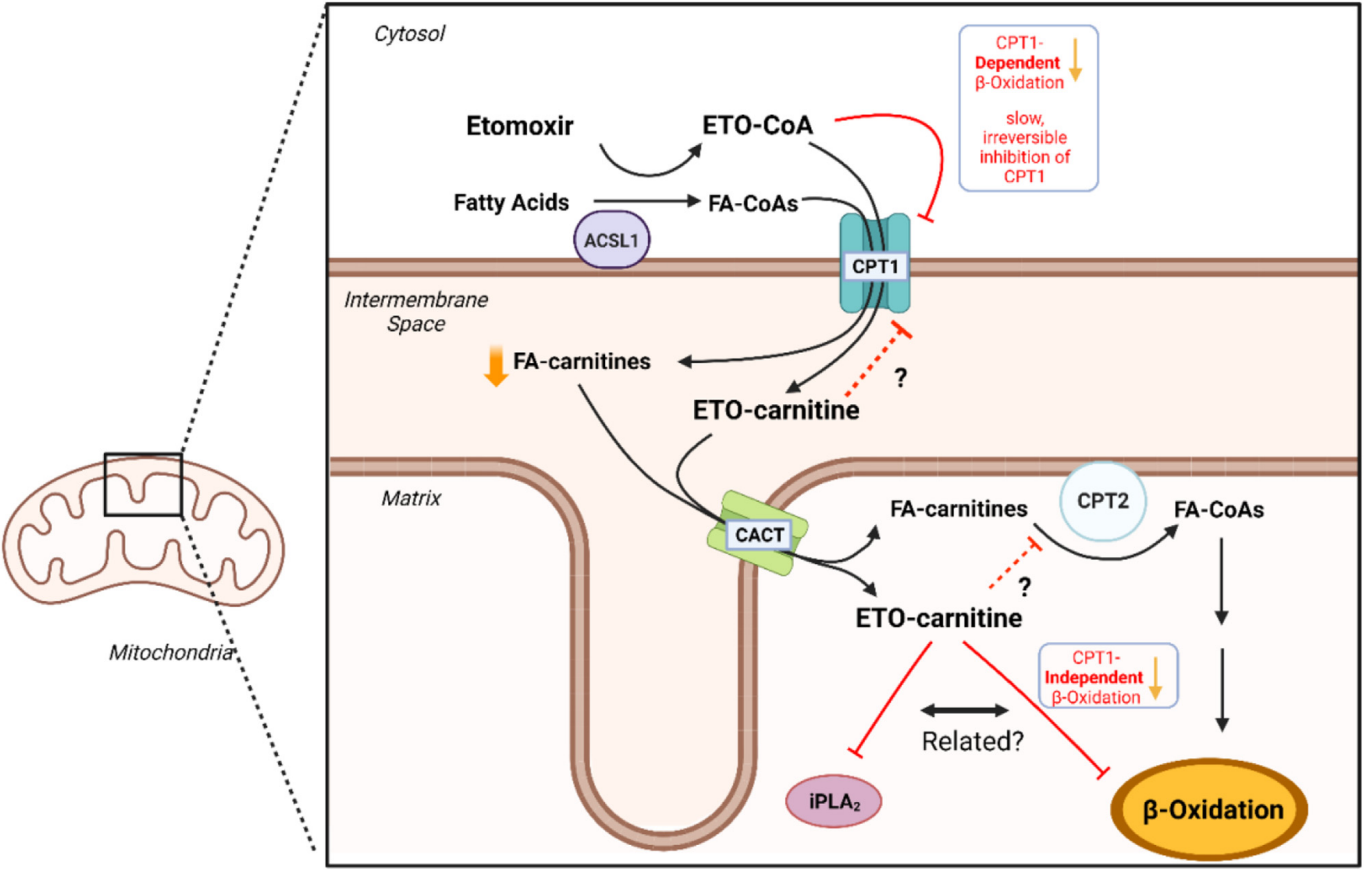
Relationship of etomoxir with the fatty acid entry pathway into mitochondria for β-oxidation. Both ETO and fatty acids are metabolized to their respective CoA metabolites, ETO-CoA and fatty acyl CoA (FA-CoA) by long chain acyl-CoA synthetase (ACSL1) in the cytosol. Carnitine palmitoyltransferase 1 (CPT1) converts FA-CoA to FA-carnitine in the rate-limiting step of β-oxidation and facilitates its transfer into the mitochondrial intermembrane space. In a novel finding, prior to irreversible inhibition of CPT1 by ETO-CoA which diminishes FA-carnitine production, CPT1 converts a fraction of ETO-CoA to ETO-carnitine. Both ETO-carnitine and FA-carnitine translocate to the mitochondrial matrix by carnitine-acylcarnitine translocase (CACT). Fatty acyl-carnitine may then be converted back into FA-CoA by the action of carnitine palmitoyltransferase 2 (CPT2) for subsequent mitochondrial β-oxidation. The Gross lab has shown that ETO-carnitine inhibits calcium-independent phospholipase A_2_ (iPLA_2_) isoforms which are known to reside in mitochondria. Also, ETO-carnitine was found to inhibit mitochondrial respiration with palmitoylcarnitine as a fuel source, indicating ETO-carnitine has a CPT1-independent inhibitory effect on mitochondrial β-oxidation, possibly involving iPLA_2_ inhibition. ETO-carnitine potentially have other off-target effects on fatty acid-processing enzymes, especially those with active site serine residues such as CPT1 or CPT2 (red dashed inhibition lines). Created with BioRender.com.

It will be interesting if this paradigm of ETO conversion to ETO-carnitine is universal for other glycidic acid derivatives such as tetradecyl glycidic acid, which possess the reactive oxirane ring and carboxylic acid moieties which could also be converted to its carnitine derivative. Tetradecyl glycidic acid has off-target effects on the renin-angiotensin system resulting in myocardial hypertrophy (10). Mechanistically, these effects remain to be understood and could have a relationship with the findings provided in this study, as other glycidic acid carnitine derivatives have not been explored in existing literature. The studies of Gross et al. also employed ST1326, an aminocarnitine analog inhibitor of CPT1, to ETO-carnitine production. ST1326 is a promising antihyperglycemic drug (11).

PLA_2_ inhibitors have broad anti-inflammatory action by preventing arachidonic acid release from phospholipid pools to be utilized in oxylipin synthesis or mitochondrial β-oxidation substrates. The high sensitivity of ETO-carnitine inhibition of iPLA_2_ contrasted to that of cPLA_2_, but still ETO-carnitine did inhibit cPLA_2_ at higher concentrations. This leads to several interesting questions that should be investigated in the future including investigating ETO-carnitine inhibition of other lipases such as endothelial lipase and sPLA_2_. Additionally, the sensitivity of iPLA_2_ to ETO-carnitine suggests it could serve as a useful chemical scaffold for the generation of a new generation of iPLA_2_ inhibitors.

ETO has been used for several decades to inhibit CPT1 and thus mitochondrial β-oxidation of fatty acids. Off-target effects of ETO have led investigators to be careful with data interpretation of ETO inhibition studies and have hampered the clinical use of ETO. The studies reported in this issue provide new insights into mechanisms responsible for the off-target effects of ETO. The discovery of ETO-carnitine was elegantly demonstrated by high resolution mass spectrometry which distinguished ETO-carnitine, 3-hydroxysteroylcarnitine, and 3-hydroxyoleoylcarnitine isobaric species. The impact of the discovery of ETO-carnitine and its diverse observed and predicted interactions with mitochondrial enzymes stimulates consideration of the wider range of potential interactions with extra-mitochondrial proteins. While these findings further solidify ETO’s unsuitable use for the specific inhibition of CPT1 and fatty acid β-oxidation, the ETO-carnitine pharmaco-metabolite could prove useful in drug development and optimization.

## Conflict of interest

The author declares that they have no conflicts of interest with the contents of this article.
